# Serum-Derived Bovine Immunoglobulin Stimulates SCFA Production by Specific Microbes in the Ex Vivo SIFR^®^ Technology

**DOI:** 10.3390/microorganisms11030659

**Published:** 2023-03-04

**Authors:** Pieter Van den Abbeele, Christopher Detzel, Alexis Rose, Stef Deyaert, Aurélien Baudot, Christopher Warner

**Affiliations:** 1 Cryptobiotix SA, Technologiepark-Zwijnaarde 82, 9052 Ghent, Belgium; 2 Proliant Health & Biologicals, LLC., Des Moines, IA 50021, USA

**Keywords:** serum-derived bovine immunoglobulin/protein isolate (SBI), ex vivo, SIFR^®^ technology, gut microbiota, dialysis, protein fermentation, prebiotic, interpersonal variation

## Abstract

Serum-derived bovine immunoglobulins (SBI) exert health benefits mediated by their ability to bind microbial components, thereby preventing translocation and subsequent inflammation. While in vivo studies have shown that a fraction of SBI also reaches the colon, little is known about the impact of SBI on the dense colonic microbiota that has great potential to impact human health. This study, therefore, investigated the impact of three bovine plasma protein fractions (SBI, bovine plasma (BP) and albumin-enriched bovine plasma (ABP)) on the gut microbiota of six human adults using the novel ex vivo SIFR^®^ technology, recently demonstrated to generate predictive findings for clinical studies. When dosed at an equivalent of 5 g/day, all protein fractions significantly increased health-related metabolites—acetate, propionate, and butyrate. Upon simulating small intestinal absorption, SBI still markedly increased acetate and propionate, demonstrating that SBI is more resistant to small intestinal digestion and absorption compared to the other protein sources. Despite noticeable interindividual differences in microbiota composition among human adults, SBI consistently stimulated a narrow spectrum of gut microbes, which largely differed from the ones that are typically involved in carbohydrate fermentation. The SBI-fermenting consortium included *B. vulgatus* and *L. edouardi* (correlating with acetate and propionate) along with *Dorea longicatena, Coprococcus comes* and the butyrate-producing bacterium SS3/4 (correlating with butyrate). Overall, this study revealed that protein bovine fractions can contribute to health benefits by specifically modulating the human gut microbiota. While health benefits could follow from the production of SCFA, a broader range of protein-derived metabolites could also be produced. This study also confirms that the concept of prebiotics (substrates selectively utilized by host microorganisms conferring a health benefit) could go beyond the use of ingestible carbohydrates and extend to partially indigestible proteins.

## 1. Introduction

Serum-derived bovine immunoglobulin/protein isolate (SBI) is a concentrated serum protein fraction containing high levels of immunoglobulins, particularly immunoglobulin G (IgG). It is generally accepted that oral immunoglobulins play a critical role in gut homeostasis by binding and neutralizing a wide array of microbial components, thereby increasing their size so they cannot cross the epithelial layer and preventing an immune response when the microbial component does pass through the epithelial layer [1]. This mechanism of action was demonstrated in a co-culture model of intestinal epithelial C2BBe1 cells and THP1 cells. In this model, the immunoglobulins in SBI could prevent the translocation of bacterial components over the epithelium, thus preventing inflammatory responses to bacterial ligands in the underlying THP1 cells [2]. The mitigating role of SBI in inflammatory responses was further demonstrated in an animal colitis model by Henderson et al. [3]. The latter study confirmed that immunoglobulins in SBI bind to antigens of enteric microbiota, thereby inhibiting the inflammatory cascades that contribute to inflammatory bowel disease (IBD). Moreover, a recent study revealed that oral administration of SBI in healthy human adults increases the levels of essential amino acids in plasma and is safe at levels as high as 20 g/day [4]. The intake of SBI can also play a protective role in immune-compromised individuals by improving intestinal barrier integrity and decreasing inflammation, as was demonstrated in HIV-infected subjects on suppressive antiretroviral therapy with chronic diarrhea [5]. Recently, SBI was even proposed as a cost-effective option to prevent progression of mild to severe COVID-19 [6]. While these studies demonstrate that the administration of SBI exerts health benefits in both healthy and immune-compromised individuals, the exact mode of action could go well beyond the binding of IgG to microbial components as reviewed by Utay et al. (2021) [6].

Today, the gut microbiota is considered among the key determinants of host physiology, both in health and disease [7,8,9]. Indeed, a broad range of physiological functions (such as strengthening the gut integrity, harvesting energy, protecting against pathogens and regulating host immunity) are—directly or indirectly—affected by gut microbes [10]. In this respect, an increased understanding of the gut microbiota and its role in human health and disease has triggered research towards modulation of the intestinal microbiota to improve human health. While carbohydrates have been studied intensively for their ability to serve as prebiotic substrates [11], microbial protein fermentation is also believed to generate a diverse range of bioactive molecules which exert host effects [12,13]. For instance, it is known that the health-beneficial propionate and butyrate are formed as products from peptide and amino acid fermentation [14]. With respect to SBI, several studies support the potential of SBI to impact the human gut microbiota. In particular, the bioactivity of IgG (i.e., the antigen-neutralizing capacity) was demonstrated in the lower gastrointestinal tract [6]. Further evidence comes from clinical data demonstrating that despite increased levels of essential amino acids in plasma (suggesting digestion and absorption), IgG was also detected in the fecal samples of the subjects consuming SBI, indicating that SBI partially escapes digestion and reaches the colon where it could alter the gut microbiota [4]. This hypothesis is supported by the observation that dietary supplementation of spray-dried porcine plasma in mice reduces mucosal inflammation by modulation of the gut microbiota [15,16]. Considering that SBI is derived from plasma, these findings highlight the potential impact of SBI on the gut microbiota. Additionally, modulation of the gut microbiome was proposed as among the modes of action of SBI in patients with COVID-19 [6]. Despite these promising findings, scientific data on the exact mechanism of action of SBI and its effects on the gut microbiota are lacking.

Clinical studies are fundamental to demonstrate health effects of test products on the host. On the other hand, they are generally less suited to unravel the mechanism of action. In particular, when modulation of the gut microbiota is expected, the inaccessibility of the site of fermentation is an important hurdle to understand the impact of test products on microbial metabolites and composition. Furthermore, the high intra- and interindividual variability in the human gut microbiota [17] highly affects the efficacy of the test product in eliciting beneficial changes via modulation of the gut microbiota [18]. Unlike clinical trials, in vitro gut models do have the potential to access the site of activity. However, the microbial composition in current short-term in vitro gut models is markedly different from the in vivo human microbiota: within 24 h of experiment, fast-growing, aerotolerant taxa represent >50% of the in vitro communities [19,20,21,22]. Similarly, long-term gut models impose highly defined conditions, thus enriching taxa that thrive in this adapted environment [23,24], within three days after inoculation of the in vivo sample [25]. Moreover, current gut models typically do not allow high-throughput studies and thus lack the capacity to address interindividual differences among human subjects or to allow a robust statistical analysis of the generated data. The recently validated ex vivo SIFR^®^ technology could mitigate some of these pitfalls [26]. First, the SIFR^®^ technology accurately preserves in vivo-derived microbiota in the lab, thus classifying the application of SIFR^®^ technology as an ex vivo study, which is a study that uses an artificial environment outside the human body with minimum alteration of natural conditions. Most importantly, using structurally different carbohydrates, findings within 24–48 h in the SIFR^®^ technology (down to species level) were shown predictive for findings of clinical studies where such carbohydrates were repeatedly administered over 2–6 weeks.

This study investigated the impact on the human adult gut microbiota of three bovine plasma protein fractions using the recently validated ex vivo SIFR^®^ technology: SBI, bovine plasma (BP) and albumin-enriched bovine plasma (ABP). Owing to the throughput of the SIFR^®^ technology, six human adult samples were included to evaluate the consistency of treatment effects of SBI, BP and ABP across different people.

## 2. Materials and Methods

### 2.1. Products

Three bovine plasma protein fractions were evaluated in the current study and provided by Proliant Health & Biologicals (Proliant Health & Biologicals LLC., Des Moines, IA, USA). Desalted bovine plasma (BP) powder has a protein content (dry basis) of 89.9%, contains 3.2% moisture and 1.67% ash. Albumin-enriched bovine plasma (ABP) powder has a protein content (dry basis) of 95.3%, contains 3.3% moisture and 5.5% ash. The third test product, serum-derived bovine immunoglobulin (SBI), contains 88.5% protein (dry basis), 0.3% moisture and 1.0% of ash.

### 2.2. Experimental Design, Timeline and Analysis

**Figure 1:**
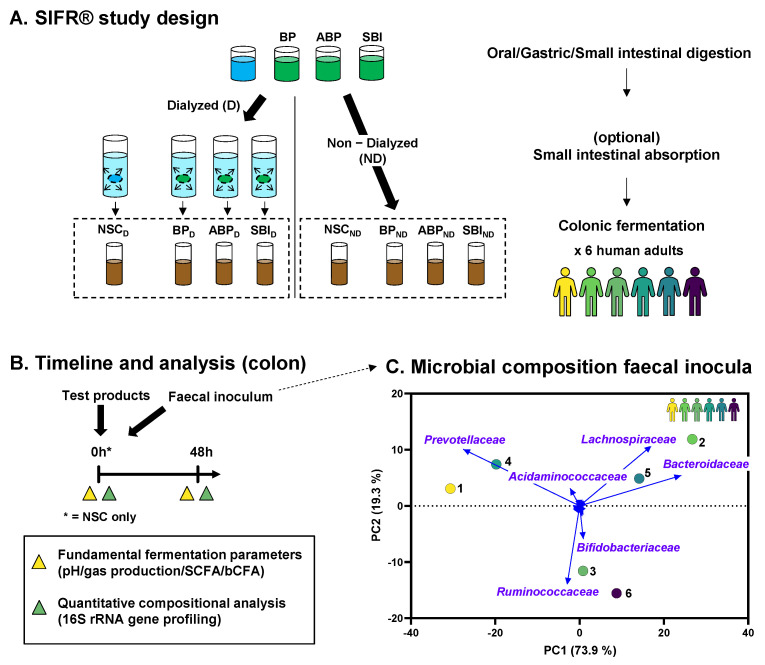
**Experimental design to study small intestinal digestion and colonic fermentation in the presence of different protein fractions (BP, ABP, and SBI) using the ex vivo SIFR^®^ technology**. (**A**) Digested protein fractions were subjected to a simulation of absorption via dialysis (D) or tested as such in 48 h colonic incubations (ND). These colonic incubations were performed to assess the fermentation of the three bovine plasma protein fractions by the human gut microbiota of human adults compared to a NSC (*n* = 6). (**B**) Sampling and analysis were performed to evaluate the effect on fundamental fermentation parameters and microbial composition. (**C**) PCA based on centered abundances at the family level (%) demonstrating the variation across the fecal microbiota of the six human adults. BP = bovine plasma, ABP = albumin-enriched bovine plasma, SBI = serum-derived Bovine Immunoglobulin, D = dialysis, ND = no dialysis, NSC = no substrate control, SCFA = short-chain fatty acid, bCFA = branched-chain fatty acid, PCA = principal component analysis, and SIFR = systemic intestinal fermentation research.

Upper gastrointestinal digestion and colonic fermentation of the bovine plasma protein fractions were investigated using the SIFR^®^ technology (Figure 1). The three products were tested at a dose equivalent to human consumption of 5 g/day. Prior to their administration to the SIFR^®^ model, the protein fractions were suspended in autoclaved distilled water at a concentration of 1% (*w*/*v*). Test products were slowly added to a beaker containing a stirring bar, after which the solution was mixed for 2 h at a speed that ensured proper mixing without foaming.

Subsequently, each test product was subjected to oral, gastric and small intestinal digestion procedures according to the latest INFOGEST 2.0 method, published by Brodkorb et al. [27]. To ensure that this method was compatible with the subsequent colonic fermentation experiments, slight modifications were implemented. These modifications include the removal of oxygen along the small intestinal incubation and simulation of the absorption process in the small intestine via the use of dialysis membranes. Dialysis was performed using 3.5 kDa membranes as previously described by Van den Abbeele et al. [28], with minor modifications to increase efficiency of removal of digested compounds: (i) dialysis was carried out over 16 h at 4 °C under and (ii) ratio of small intestinal content over dialysis fluid was 1:20. During the dialysis procedure, digested protein fractions (such as amino acids and short peptides) moved from the intestinal content to the dialysis solution via passive diffusion whereas the relevant fraction of the test products (>3.5 kDa) was retained and thus part of the colonic medium at the start of the colonic incubations.

Colonic incubations were performed as described recently [26]. Briefly, individual bioreactors were processed in parallel in a bioreactor management device (Cryptobiotix, Ghent, Belgium). Each bioreactor contained 5 mL of nutritional medium-fecal inoculum blend supplemented with digested test products derived from the simulated small intestinal digestion protocol, then sealed individually, before being rendered anaerobic. Blend M0003 was used for preparation of the nutritional medium (Cryptobiotix, Ghent, Belgium). After preparation, bioreactors were incubated under continuous agitation (140 rpm) at 37 °C for 48 h (MaxQ 6000, Thermo Scientific, Thermo Fisher Scientific, Merelbeke, Belgium). Upon gas pressure measurement in the headspace, liquid samples were collected for subsequent analysis.

Fresh fecal samples were collected according to a procedure approved by Ethics Committee of the University Hospital Ghent (reference number BC-09977). This procedure required participants to sign an informed consent in which they donate their fecal sample for the current study. The selection criteria for the six donor samples used herein were as follows: 25–65 years of age, no antibiotic use in the past 3 months, no gastro-intestinal disorders (cancer, ulcers, IBD), no use of probiotic, non-smoking, alcohol consumption < 3 units/d and BMI < 30. For this specific study, four male and two female donor samples were tested. The age of the test subjects ranged from 27 to 39 years and was on average 33.8 years. 

For each of the six fecal microbiota, a no substrate control (NSC) was initiated simultaneously, consisting of background medium and microbiota without test product. The advantage of comparing test products to such NSC is that any changes observed between the NSC and test products can solely be attributed to the addition of the test products. 

### 2.3. Fundamental Fermentation Parameters 

The level of acidification during the short-term incubations is a measure for the degree of bacterial fermentation activity. The pH was measured immediately using an electrode (Hannah Instruments Edge HI2002, Temse, Belgium). As the incubations were performed in a closed incubation system, one could determine the accumulation of gases in the headspace by penetrating the rubber septum with a needle connected to a pressure meter. Further, short-chain fatty acids (SCFA) and branched-chain fatty acids (bCFA) were quantified by gas chromatography (GC) coupled to flame ionization detection (Trace 1300, Thermo Fisher Scientific, Merelbeke, Belgium), upon diethyl ether extraction as previously described [29]. All these endpoints do not determine the instantaneous microbial activity, yet they reflect the activity during the preceding incubation period. 

### 2.4. Microbial Composition Analysis

Quantitative data were obtained by correcting abundances (%; 16S rRNA gene profiling) with total cell counts for each sample (cells/mL; flow cytometry), resulting in estimated cell counts/mL of different taxonomic groups. Initially, a bacterial cell pellet was obtained by centrifugation of 1 mL sample for 5 min at 9000× *g*. DNA was extracted via the SPINeasy DNA Kit for Soil (MP Biomedicals, Eschwege, Germany), according to manufacturer’s instructions. Upon DNA extraction, library preparation and sequencing were performed on an Illumina MiSeq platform with v3 chemistry. The 16S rRNA gene V3-V4 hypervariable regions were amplified using primers 341F (50 -CCT ACG GGN GGC WGC AG-30) and 785Rmod (50 -GAC TAC HVG GGT ATC TAA KCC-30). Results were analyzed at different taxonomic levels (phylum, family and OTU (operational taxonomic unit) level). For total cell count analysis, liquid samples were diluted in anaerobic phosphate-buffered saline (PBS), after which cells were stained with SYTO 16 at a final concentration of 1 µM and counted via a BD FACS Verse flow cytometer (BD, Erembodegem, Belgium). Data were analyzed using FlowJo, version 10.8.1.

### 2.5. Statistics

Univariate and multivariate analyses were performed by GraphPad Prism (v9.3.1; www.graphpad.com, accessed on 28 January 2023), while Regularized Canonical Correlation Analysis (rCCA) was executed using the mixOmics package with the shrinkage method for estimation of penalization parameters (version 6.16.3) in R (4.1.1; www.r-project.org; accessed on 26 December 2022) [30]. Treatment effects were assessed using repeated measures ANOVA analysis (based on paired testing considering six donors in *n* = 1) and the Benjamini–Hochberg correction was applied to *p*-values [31]. Statistical differences between treatments and the blank are indicated with * (0.01 < *p* < 0.05), ** (0.001 < *p* < 0.01) or *** (*p* < 0.001), while differences between protein fractions ABP/SBI and fraction BP are indicated with $/$$/$$$. For analysis of microbial composition, three measures were taken. First, the aforementioned statistical analysis was performed on the log_10_-transformed values. Second, a value of a given taxonomic group below the limit of detection (LOD) was considered equal to the overall LOD according to the procedure described previously [26]. Finally, a threshold was set in order to retain the 100 most abundant OTUs in the analysis, to avoid excessive *p*-values corrections.

## 3. Results

### 3.1. Microbiota of Six Human Adults Cover Interpersonal Differences in Gut Microbiota Composition

The microbial composition of the fecal microbiota (used to inoculate SIFR^®^ reactors) was markedly different between the six human adults tested (Figure 1C). Most of the variation was explained along PC1 (explaining 73.9% variation) and differentiated between donors with high *Prevotellaceae* (left: donors 1/4) and donors with high *Bacteroidaceae* levels (right: donors 2/3/5/6). The stratification of donors according to these two families is in line with the concept stratifying human adult microbiota according to gut enterotypes [32], suggesting that the six human adults included in the current study covered a spectrum of microbiota composition that occurs across the human adult population. 

### 3.2. Bovine Plasma Protein Fractions Stimulated Growth of Human Adult Gut Microbiota Ex Vivo 

**Figure 2:**
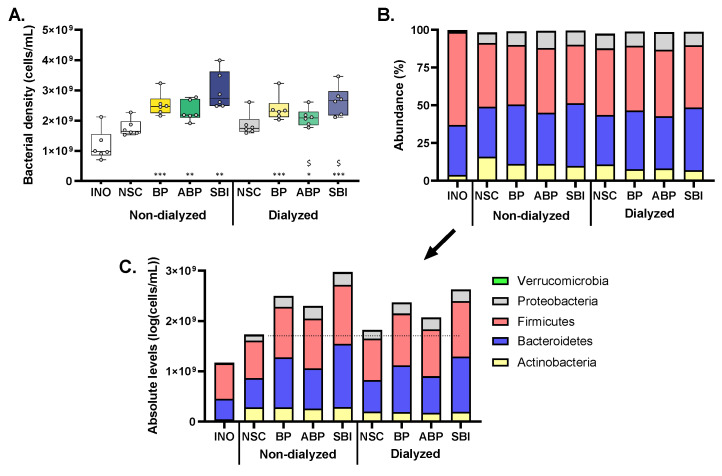
**Three bovine plasma protein fractions (BP, ABP and SBI) stimulated growth of human adult gut microbiota ex vivo.** (**A**) Bacterial cell density of microbial communities derived from human adults (*n* = 6) as tested via the ex vivo SIFR^®^ technology (cells/mL). Three protein bovine plasma fractions (BP/ABP/SBI) were tested at a dose equivalent of 5 g/day, with or without prior dialysis, simulating absorption in the small intestine, compared to a NSC. Samples were collected after 0 h (INO) and 48 h of simulated colonic incubations. Statistical differences between treatments and the NSC are indicated with * (0.01 < *p_adjusted_* < 0.05), ** (0.001 < *p_adjusted_* < 0.01) or *** ( *p_adjusted_* < 0.001), while differences between protein fractions ABP/SBI and fraction BP are indicated with $ (0.01 < *p_adjusted_* < 0.05). The microbial composition (phylum level) in samples collected after 0 h (INO) and 48 h of simulated colonic incubations, presented both as (**B**) proportional (%) and (**C**) absolute values (cells/mL), as averaged across the six human adults tested. BP = bovine plasma, ABP = albumin-enriched bovine plasma, SBI = serum-derived bovine immunoglobulin, SIFR = systemic intestinal fermentation research, and NSC = no substrate control.

Bacterial cell density was analyzed by flow cytometry (Figure 2). Results revealed a marked increase in cell counts between 0 h (INO) and 48 h samples, reflecting the growth of the ex vivo microbial communities during SIFR^®^ incubations. The three bovine plasma protein fractions further significantly increased cell numbers versus the NSC at 48 h, indicating that the gut microbiota was able to use the bovine protein fractions as substrates for growth. Interestingly, SBI more profoundly increased cell numbers as compared to BP and ABP, especially upon dialysis when the increase upon SBI treatment was statistically significant compared to BP.

As a remark, given the marked differences in bacterial cell numbers across the samples, the proportional data obtained via sequencing (%; Figure 2B) was corrected for total cell counts to obtain insights in the true changes in microbial composition upon treatment (Figure 2C). Subsequent analysis of microbial composition is thus solely based on quantitative insights in microbial composition.

### 3.3. Bovine Plasma Protein Fractions Supported a High Microbial Diversity

Four diversity indices were calculated to obtain optimal insights in microbial diversity (Figure S1). First, the observed number of operational taxonomic unit (OTUs) and the Chao1 diversity index were calculated as a measure of species richness. Further, two additional measures were based on both species’ richness and evenness (the reciprocal Simpson diversity index and the Shannon diversity index). These indices are rather based on the dominant community members and have higher values as the dominant members are more evenly distributed.

All four indices suggested a similar microbial diversity for the NSC at 48 h as compared to the initial inocula (INO). The increase in cell numbers during the 48 h incubations was thus due to the growth of a diverse range of bacteria, confirming that the SIFR^®^ technology allows for the growth of a broad range of in vivo-derived gut microbes.

All treatments maintained a similar microbial diversity compared to the NSC when focusing on species richness (Figure S1A,B). When also accounting for species evenness (Figure S1C,D), microbial diversity tended to slightly decrease compared to the NSC, reflecting a less even distribution among the dominant gut microbes upon treatment, thus suggesting that the bovine protein fractions are selectively utilized by specific gut micro-organisms. Such decrease in diversity has been observed upon prebiotic intervention [33] and is in line with the prebiotic definition that defines prebiotics as substrates that are selectively utilized by specific micro-organisms [34].

### 3.4. Bovine Plasma Protein Fractions Stimulated Firmicutes and Particularly Bacteroidetes 

**Figure 3:**
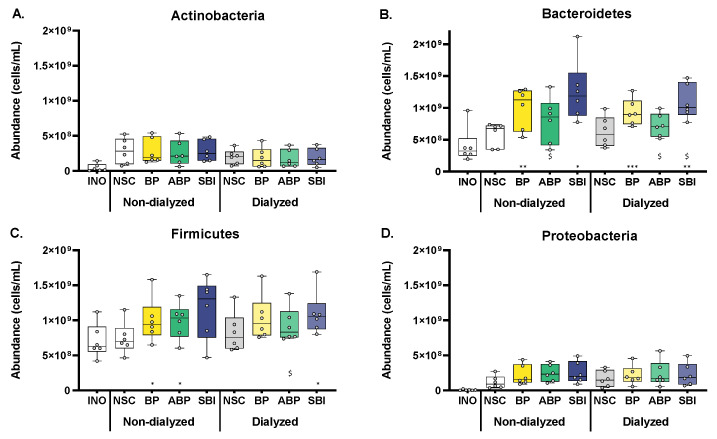
**Three bovine plasma protein fractions (BP, ABP and SBI) stimulated Firmicutes and particularly Bacteroidetes members of the human adult gut microbiota ex vivo.** The impact of three bovine plasma protein fractions, dosed at an equivalent of 5 g/day, with or without prior dialysis as a simulation of small intestinal absorption, compared to the NSC, on the abundance of (**A**) Actinobacteria, (**B**) Bacteroidetes, (**C**) Firmicutes and (**D**) Proteobacteria, as tested via the SIFR^®^ technology for human adults (*n* = 6). Samples were collected after 0 h (INO) and 48 h of simulated colonic incubations. Statistical differences between treatments and the NSC are indicated with * (0.01 < *p_adjusted_* < 0.05), ** (0.001 < *p_adjusted_* < 0.01) or *** (*p_adjusted_* < 0.001), while differences between protein fractions ABP/SBI and fraction BP are indicated with $ (0.01 < *p_adjusted_* < 0.05). BP = bovine plasma, ABP = albumin-enriched bovine plasma, SBI = serum-derived bovine immunoglobulin, SIFR = systemic intestinal fermentation research, and NSC = no substrate control.

The four most abundant phyla were Actinobacteria, Bacteroidetes, Firmicutes and Proteobacteria (Figure 2B,C). For each of these phyla, an in-depth analysis was performed (Figure 3). Both the dialyzed and non-dialyzed bovine fractions BP and SBI significantly increased Bacteroidetes levels, with the increases being most profound for SBI. All protein fractions tended to increase Firmicutes levels as well, with some differences being significant. In particular for the dialyzed fractions, the increase in Firmicutes levels for SBI and BP was more pronounced as compared to the ABP. 

### 3.5. Bovine Plasma Protein Fractions Stimulated Specific Gut Microbes Ex Vivo 

**Figure 4:**
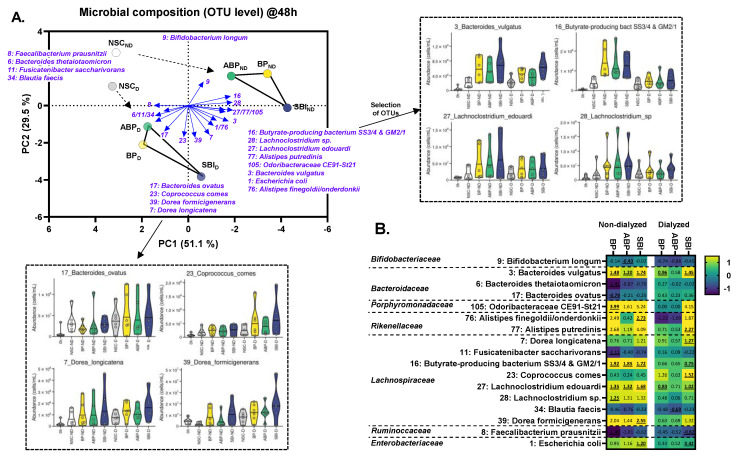
**Three bovine plasma protein fractions (BP, ABP and SBI) stimulated specific human adult gut microbes ex vivo.** (**A**) PCA summarizing the impact on the gut microbiota of three bovine plasma protein fractions (BP/ABP/SBI), dosed at an equivalent of 5 g/day, with or without prior dialysis as a simulation of small intestinal absorption, compared to the NSC, as tested via the SIFR^®^ technology for human adults (*n* = 6). The PCA was based on the standardized abundances of significantly affected OTUs by any of the treatments (FDR = 0.20) as quantified via 16S rRNA gene sequencing combined with flow cytometry (cells/mL), at 48 h after initiation of colonic incubation. The different OTUs that underly this clustering are shown by the blue arrows. In the dotted frames, a detailed representation of the OTUs that significantly increased or decreased upon treatment with the test products is shown. (**B**) Heatmap demonstrating the impact of the bovine plasma protein fractions on OTUs that were significantly affected by any of the treatments (FDR = 0.20), expressed as log_2_ (treatment/NSC), averaged over six human adults at 48 h. Significant differences compared to the NSC are indicated by bold and underlining. BP = bovine plasma, ABP = albumin-enriched bovine plasma, SBI = serum-derived bovine immunoglobulin, PCA = principal component analysis, SIFR = systemic intestinal fermentation research, NSC = no substrate control, and OTU = operational taxonomic unit.

To evaluate the changes in microbial composition at higher taxonomic resolution, both an exploratory (Figure 4A) and in-depth statistical analysis (Figure 4B) was performed at OTU level. Interestingly, 17 OTUs were significantly affected (FDR = 0.20). These OTUs were abundant as they covered, on average, 51 ± 5% of a given sample.

The non-dialyzed protein fractions resulted in a marked shift along PC1 in the PCA, suggesting a marked treatment effect on OTUs related to the butyrate-producing bacterium SS3/4 (OTU16), *Lachnoclostridium* sp. (OTU27/28), *Alistipes* sp. (OTU76/77), *Escherichia coli* (OTU1), and *Bacteroides vulgatus* (OTU3). Further, the dialyzed fractions resulted in a more attenuated shift along PC1, suggesting a similar, yet attenuated stimulation of aforementioned taxa. Interestingly, there was an additional downward shift along PC2, suggesting the specific stimulation of microbial taxa that were likely better adapted to ferment non-dialyzed (and less digestible protein fractions), i.e., *Dorea* sp. (OTU7/39), *Coprococcus comes* (OTU23) and *Bacteroides ovatus* (OTU17). The increase in the *Coprococcus comes*-related OTU was most pronounced and significant for SBI, which was of particular interest given the concomitant decrease in *Blautia* sp. (OTU36). 

### 3.6. Bovine Plasma Protein Fractions, Particularly SBI, Boosted Production of Health-Related SCFA

**Figure 5:**
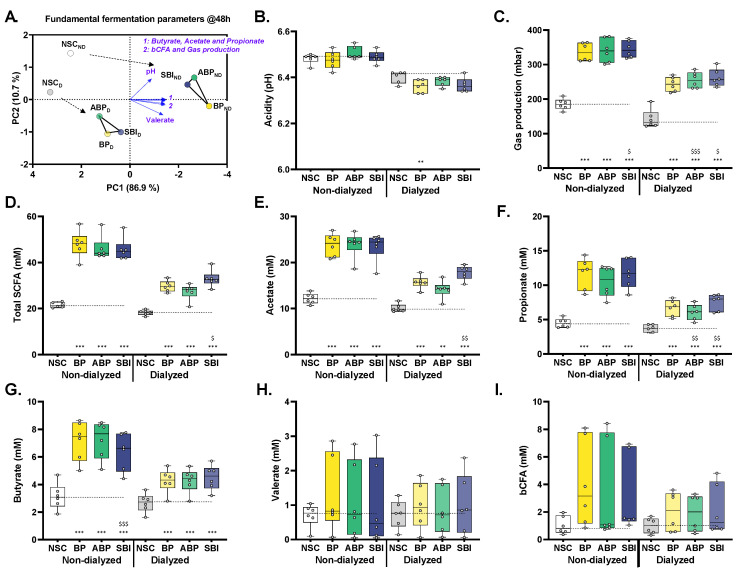
**Three bovine plasma protein fractions (BP, ABP and SBI), particularly SBI, boosted production of health-related SCFA by human adult gut microbes ex vivo.** (**A**) PCA analysis based on the standardized average values across donors (*n* = 6) for fundamental fermentation parameters (pH, gas production, acetate, propionate, butyrate, valerate and bCFA) upon treatment with three bovine plasma protein fractions (BP/ABP/SBI), dosed at an equivalent of 5 g/day, with or without prior dialysis as a simulation of small intestinal absorption, compared to a NSC, as tested via the SIFR^®^ technology. The impact on (**B**) pH, (**C**) gas production, (**D**) total SCFA, (**E**) acetate, (**F**) propionate, (**G**) butyrate, (**H**) valerate and (**I**) bCFA for all test products compared to the NSC. Statistical differences between treatments and the NSC are indicated with ** (0.001 < *p* < 0.01) or *** (*p* < 0.001), while differences between protein fractions ABP/SBI and fraction BP are indicated with $/$$/$$$. BP = bovine plasma, ABP = albumin-enriched bovine plasma, SBI = serum-derived bovine immunoglobulin, PCA = principal component analysis, SIFR = systemic intestinal fermentation research, NSC = no substrate control, SCFA = short-chain fatty acid, and bCFA = branched-chain fatty acid.

To investigate product-specific treatment effects on microbial metabolite production, fundamental fermentation parameters were recorded at 48 h. A first exploratory PCA analysis demonstrated that the test products positioned distinctly different from the NSC along PC1 that related with higher acetate, propionate, butyrate and valerate levels (Figure 5A). All bovine protein fractions (dialyzed and non-dialyzed) significantly increased acetate, propionate, butyrate and thus also total SCFA levels compared to the NSC, with the extent of the changes being lower for the dialyzed fractions (Figure 5C–G). The large standard deviations for valerate and bCFA production reflects interpersonal differences among the six human adults tested (Figure 5H,I). Interestingly, the two donors with high initial *Prevotellaceae* levels (donors 1/4; Figure 1C) were the two donors for which high valerate levels were observed. While the three non-dialyzed bovine protein fractions exerted similar effects, product-specific effects were observed for the dialyzed test products. Upon dialysis, SBI resulted in significantly higher acetate, propionate and thus also total SCFA production compared to BP (Figure 5D–F), suggesting that SBI is more resistant to small intestinal digestion and absorption compared to BP and ABP.

Finally, the production of SCFA markedly correlated with the stimulation of specific OTUs (Figure S2). For example, the production of butyrate correlated to the presence of OTU16 related to the butyrate-producing bacterium SS3/4. Further, acetate and propionate production correlated strongly with, amongst others, *Bacteroides vulgatus* (OTU3) and *Lachnoclostridium edouardi* (OTU27).

## 4. Discussion

The present study investigated colonic fermentation of three bovine plasma protein fractions by human adult gut microbes using the ex vivo SIFR^®^ technology. The biorelevance of this technology followed from the growth of a diverse, in vivo-like microbiota along the entire duration of the colonic incubations. Interestingly, all three protein bovine fractions increased the total number of microbial cells, while maintaining a similar diversity as compared to the untreated parallel test arm (NSC). Further, all bovine plasma protein fractions markedly increased the production of health-related metabolites (e.g., SCFA), while significantly stimulating a narrow spectrum of OTUs related to gut microbes that likely constitute a consortium that is specialized in fermenting bovine plasma proteins. These effects were particularly pronounced for SBI and to a lesser extent BP.

Colonic fermentation of the bovine plasma protein fractions was tested both with and without prior simulated small intestinal absorption by means of a dialysis approach. The additional implementation of small intestinal absorption resulted in attenuated, yet similar effects on SCFA and specific gut microbes. This result was anticipated given the removal of amino acids and small peptides by such dialysis approach. Interestingly, OTUs related to *Bacteroides ovatus*, two *Dorea* species and *Coprococcus comes* markedly increased upon administration of the dialyzed protein fractions, suggesting that these species thrive on protein fractions that are more resistant to digestion and absorption. In contrast, the non-dialyzed protein fractions (i.e., containing more amino acids and short peptides) markedly increased OTU16, related to a major butyrate-producing member of the human gut microbiota (i.e., butyrate-producing bacterium SS3/4) [35], suggesting that this species is likely specialized in producing butyrate particularly from amino acids and shorter peptides derived from bovine plasma proteins. These observations underline the need for a simulation of the small intestinal absorption to obtain more biorelevant data. The subsequent discussion will thus focus on data obtained with the dialyzed test products.

When tested at a dose equivalent to 5 g/day, all three bovine protein fractions significantly increased the production of acetate, propionate and butyrate as compared to the untreated test condition (NSC). Upon simulating small intestinal absorption, it was particularly SBI that most strongly increased acetate, propionate and butyrate levels. Acetate, propionate and butyrate are SCFA that have each been related with particular health benefits as reviewed by Rivière et al. [36]. Briefly, in addition to exerting anti-inflammatory effects, SCFA also decrease the pH of the colonic lumen which increases mineral absorption and inhibits growth of pathogens. Acetate is a minor energy source for epithelial cells but reaches the portal vein after which it is metabolized in various tissues. Like acetate, propionate reaches the portal vein after which it is taken up by the liver. Health effects related to propionate include that it promotes satiety, lowers blood cholesterol, decreases liver lipogenesis and improves insulin sensitivity. Butyrate on the other hand is the preferred energy source of epithelial cells and plays a protective role against colon cancer and colitis. The three bovine protein fractions and particularly SBI could thus exert health benefits via the stimulation of SCFA production.

Upon implementation of small intestinal absorption, SBI still markedly stimulated a specific consortium of gut microbes consisting of OTUs related to *Bacteroides vulgatus, Lachnoclostridium edouardi, Dorea longicatena, Coprococcus comes* and the butyrate-producing bacterium SS3/4 (Figure 5B). The OTUs related to *B. vulgatus* and *L. edouardi* are correlated with acetate and propionate production (Figure S2). *Bacteroides* and *Lachnoclostridium* species are indeed known to ferment proteins [37], with a long-term diet rich in protein even having been associated with the *Bacteroides* enterotype (i.e., a microbiota strongly enriched with *Bacteroides* species) [38]. This correlation stresses the potential important contribution of *Bacteroides* species to protein fermentation. *Bacteroides* species produce acetate and propionate as main end metabolites which is thus in line with the observed correlations [14]. Further, the OTUs related to *Dorea longicatena, Coprococcus comes* and the butyrate-producing bacterium SS3/4 rather related with butyrate production. Both *Coprococcus comes* and the butyrate-producing bacterium SS3/4 are indeed amongst the major butyrate producers in the gut [35]. While *Coprococcus comes* produces butyrate via the butyrate kinase pathway, the butyrate-producing bacterium SS3/4 uses the butyryl-coA:acetate CoA-transferase gene [14].The increase in the *Coprococcus comes*-related OTU was most pronounced for SBI and was of particular interest given the concomitant decrease in *Blautia* sp. (OTU36) (Figure 5B). Such shift from *Blautia* towards *Coprococcus* has been linked with increased levels of indole-propionic acid (IPA) [39], a deamination product of tryptophan metabolism with health-promoting effects. The health-promoting effects of IPA were demonstrated in various recent studies focusing on type 2 diabetes [40], host gut barrier function and antioxidant activity [41], the gut–brain axis [42] and non-alcoholic steatohepatitis [43]. Overall, this study revealed the existence of a consortium of gut microbes specialized in the fermentation of bovine plasma proteins. The strong correlation of these species with specific SCFA suggests their involvement in the production of these health-related metabolites upon intake of bovine plasma proteins. Interestingly, this specific microbial consortium is different from the one that is typically impacted by carbohydrate fermentation.

Addressing interindividual differences among human subjects is a prerequisite to ensure representative outcomes in gastrointestinal research, given the interpersonal differences in gut microbiota [17] and how these differences impact the response to interventions [18]. The intrinsically high throughput of the SIFR^®^ technology allowed for the inclusion of samples from six human adults in the study design. Indeed, marked interpersonal differences were observed upon analyzing the microbial composition of the fecal inocula (Figure 1). While the treatment effects were relatively consistent across the six adult donor samples tested, different response scenarios were noted in terms of valerate and bCFA production. These metabolites were particularly produced by the microbiota of the two donors with initial high levels of *Prevotellaceae* (donors ¼), suggesting that treatment responses to administration of bovine plasma protein fractions could relate with the concept of enterotypes [38,44]. However, considering the relatively low number of donor samples included in this study, it will be important to confirm these findings in future studies with a larger cohort. 

## 5. Conclusions

While the main mode of action of SBI relies on binding antigens to prevent inflammation [2,3,4], the current study demonstrated that SBI can also exert health benefits via modulation of the human adult gut microbiota. SBI was shown to be specifically fermented by a narrow consortium of gut microbes that related with the production of specific health-related SCFA. In other words, this exploratory study demonstrated the prebiotic potential of protein bovine fractions, particularly SBI, given that a recent international scientific consensus now defines prebiotics as substrates that are selectively utilized by host microorganisms, thus conferring a health benefit [34]. While the aspect of selective utilization was demonstrated in this study, the health benefits of protein bovine fractions could follow from the production of health-related SCFA. Moreover, protein-based products could contribute to the production of protein-derived metabolites with particular health benefits, such as GABA (related to the gut–brain axis) and IPA (related to immune heath and T2D). In this regard, the specific stimulation of *Coprococcus comes* (previously linked with IPA production) by SBI as demonstrated in this study could be an interesting route for further research. The prebiotic potential of the bovine protein fractions also highlights that the concept of prebiotics could go beyond the use of indigestible carbohydrates. 

Overall, our findings support further research into the production of protein-derived metabolites and their health benefits. Given the interindividual differences that were observed in this study for specific metabolites (valerate and bCFA), such studies should include a broad range of donors to account for interindividual differences in gut microbiota composition and the response to treatments.

## Data Availability

The datasets generated during and/or analyzed during the current study are available from the corresponding author with approval from funding source upon reasonable request.

## Supplementary Materials

The following are available online at https://www.mdpi.com/article/10.3390/microorganisms11030659/s1, Figure S1: Three bovine plasma protein fractions (BP, ABP and SBI) supported a high microbial diversity of the human adult gut microbiota ex vivo; Figure S2: The production of SCFA markedly correlated with the presence of specific OTUs.
