# Supplementary material for: Serum-Derived Bovine Immunoglobulin Stimulates SCFA Production by Specific Microbes in the Ex Vivo SIFR® Technology
Source: Microorganisms. 2023 Mar 4;11(3):659. doi: 10.3390/microorganisms11030659 (PMC10053870; doi:10.3390/microorganisms11030659)
Supplement: Supplementary file 1 [file microorganisms-11-00659-s001.zip › microorganisms-2216395-supplementary.pdf]

microorganisms

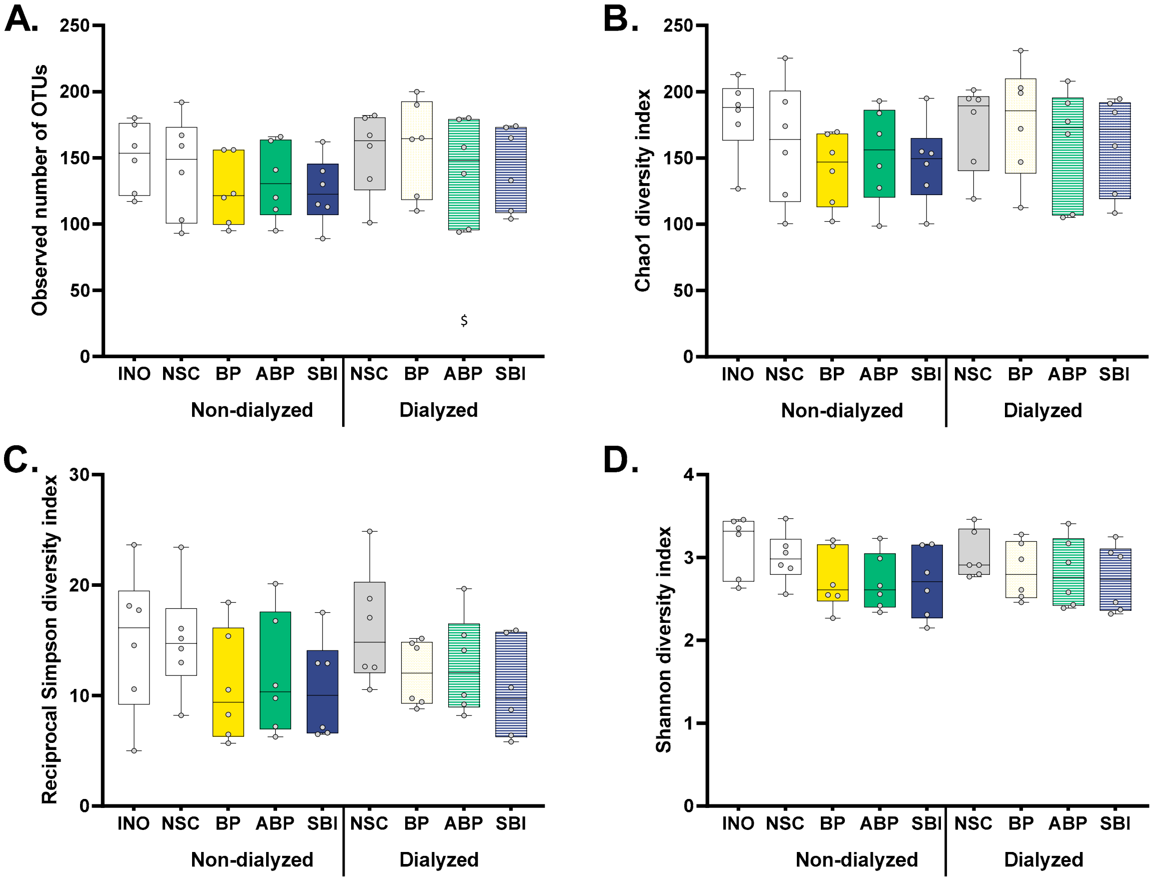

Figure S1. Three bovine plasma protein fractions (BP, ABP and SBI) supported a high microbial diversity of the human adult gut microbiota *ex vivo*. The impact on the observed number of OTUs (**A**), the Chao1 diversity index (**B**), the reciprocal Simpson diversity index (**C**) and the Shannon diversity index (**D**) for three bovine plasma protein fractions, dosed at an equivalent of 5 g/d, with or without preceding dialysis as a simulation of small intestinal absorption, compared to a NSC, as tested via the SIFR® technology for human adults (n = 6). Samples were collected after 0h (INO) and after 48h of simulated colonic incubations. Statistical differences between treatments and the NSC are indicated with * ($0.01 < p < 0.05$), ** ($0.001 < p < 0.01$) or *** ($p < 0.001$), while differences between protein fractions ABP/SBI and fraction BP are indicated with $/$$/$$$. BP = Bovine Plasma, ABP = Albumin-enriched Bovine Plasma, SBI = Serum-derived Bovine Immunoglobulin, SIFR = Systemic Intestinal Fermentation Research, NSC = no substrate control, OTU = Operational taxonomic unit.

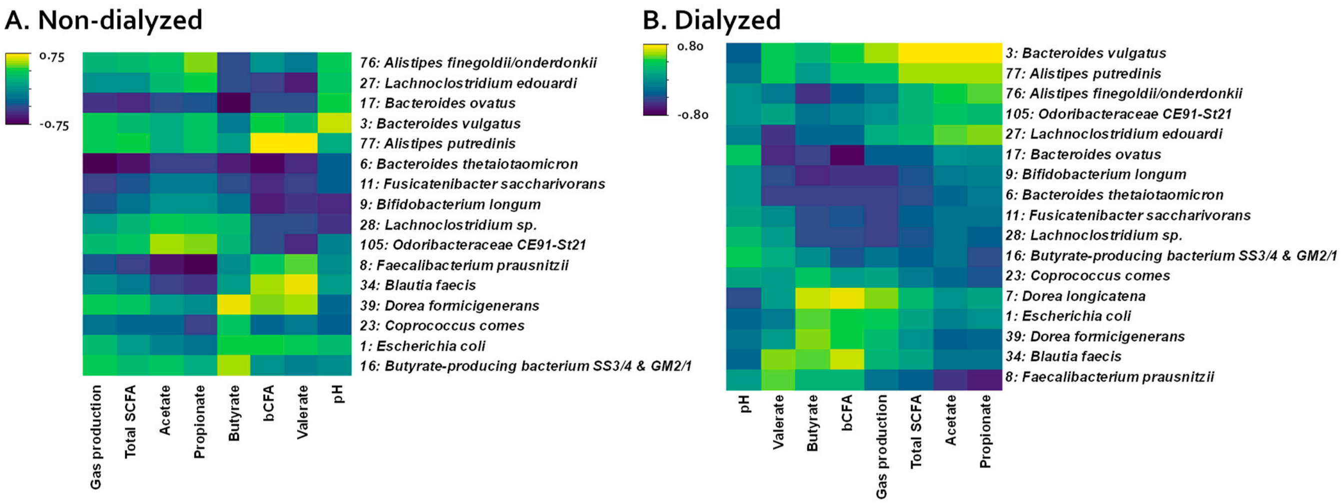

Figure S2. The production of SCFA markedly correlated with the presence of specific OTUs. rCCA summarizing the correlations between fundamental fermentation parameters and compositional data for samples collected at 48h after initiation of the colonic incubations, both for the non-dialyzed (**A**) and dialyzed (**B**) plasma protein fractions, averaged over simulations for six human adults using the SIFR® technology. rCCA = Regularized Canonical Correlation Analysis , SIFR = Systemic Intestinal Fermentation Research.
